# Histone 2A Family Member J Drives Mesenchymal Transition and Temozolomide Resistance in Glioblastoma Multiforme

**DOI:** 10.3390/cancers12010098

**Published:** 2019-12-30

**Authors:** Hsun-Hua Lee, Che-Hsuan Lin, Hui-Yu Lin, Chia-Hao Kuei, Jing-Quan Zheng, Yuan-Hung Wang, Long-Sheng Lu, Fei-Peng Lee, Chaur-Jong Hu, Dean Wu, Yuan-Feng Lin

**Affiliations:** 1 Graduate Institute of Clinical Medicine, College of Medicine, Taipei Medical University, Taipei 11031, Taiwan; kaorulei@yahoo.com.tw (H.-H.L.); candycarol0227@gmail.com (H.-Y.L.); pplay1028@gmail.com (C.-H.K.); 16044@s.tmu.edu.tw (J.-Q.Z.); d508091002@tmu.edu.tw (Y.-H.W.); chaurjongh@tmu.edu.tw (C.-J.H.); 2 Department of Neurology, School of Medicine, College of Medicine, Taipei Medical University, Taipei 11031, Taiwan; 3 Dizziness and Balance Disorder Center, Shuang-Ho Hospital, Taipei Medical University, New Taipei City 23561, Taiwan; 4 Department of Neurology, Shuang-Ho Hospital, Taipei Medical University, New Taipei City 23561, Taiwan; 5 Taipei Neuroscience Institute, Taipei Medical University, New Taipei City 23561, Taiwan; 6 Graduate Institute of Medical Sciences, College of Medicine, Taipei Medical University, Taipei 11031, Taiwan; cloudfrank@gmail.com; 7 Department of Otolaryngology, Taipei Medical University Hospital, Taipei Medical University, Taipei 11031, Taiwan; 8 Breast Center, Department of General Surgery, Cardinal Tien Hospital, Xindian District, New Taipei City 231, Taiwan; 9 Urology, Division of Surgery, Cardinal Tien Hospital, Xindian District, New Taipei City 231, Taiwan; 10 Department of Critical Care Medicine, Shuang Ho Hospital, Taipei Medical University, New Taipei City 23561, Taiwan; 11 Department of Medical Research, Shuang-Ho Hospital, Taipei Medical University, New Taipei City 23561, Taiwan; 12 Department of Radiation Oncology, TMU Hospital, Taipei Medical University, Taipei 11031, Taiwan; lslu@tmu.edu.tw; 13 Department of Otolaryngology, Shuang-Ho Hospital, Taipei Medical University, New Taipei City 23561, Taiwan; fplee@tmu.edu.tw; 14 Department of Otolaryngology, School of Medicine, College of Medicine, Taipei Medical University, Taipei 11031, Taiwan; 15 Sleep Center, Shuang-Ho Hospital, Taipei Medical University, New Taipei City 23561, Taiwan; 16 Cell Physiology and Molecular Image Research Center, Wan Fang Hospital, Taipei Medical University, Taipei 116, Taiwan

**Keywords:** GBM, Temozolomide, *H2AFJ*, *MGMT*, precision medicine

## Abstract

Glioblastoma multiforme (GBM) is the most aggressive brain tumor and has a poor prognosis and is poorly sensitive to radiotherapy or temozolomide (TMZ) chemotherapy. Therefore, identifying new biomarkers to predict therapeutic responses of GBM is urgently needed. By using The Cancer Genome Atlas (TCGA) database, we found that the upregulation of histone 2A family member J (H2AFJ), but not other H2AFs, is extensively detected in the therapeutic-insensitive mesenchymal, IDH wildtype, MGMT unmethylated, or non-G-CIMP GBM and is associated with poor TMZ responsiveness independent of radiation. Similar views were also found in GBM cell lines. Whereas H2AFJ knockdown diminished TMZ resistance, H2AFJ overexpression promoted TMZ resistance in a panel of GBM cell lines. Gene set enrichment analysis (GSEA) revealed that H2AFJ upregulation accompanied by the activation of TNF-α/NF-κB and IL-6/STAT3-related pathways is highly predicted. Luciferase-based promoter activity assay further validated that the activities of NF-κB and STAT3 are causally affected by H2AFJ expression in GBM cells. Moreover, we found that therapeutic targeting HADC3 by tacedinaline or NF-κB by ML029 is likely able to overcome the TMZ resistance in GBM cells with H2AFJ upregulation. Significantly, the GBM cohorts harboring a high-level H2AFJ transcript combined with high-level expression of TNF-α/NF-κB geneset, IL-6/STAT3 geneset or HADC3 were associated with a shorter time to tumor repopulation after initial treatment with TMZ. These findings not only provide H2AFJ as a biomarker to predict TMZ therapeutic effectiveness but also suggest a new strategy to combat TMZ-insensitive GBM by targeting the interaction network constructed by TNF-α/NF-κB, IL-6/STAT3, HDAC3, and H2AFJ.

## 1. Introduction

Glioblastoma multiforme (GBM) is the most aggressive brain tumor and is a grade IV histological malignancy, according to the WHO classification [1]. Patients with GBM have a poor prognosis, reported as only a 4–5% five-year survival rate [2,3] and a median survival time of approximately 14–15 months from diagnosis, probably owing to the poor response to radiotherapy and chemotherapy [4,5]. Based on molecular signatures, GBM has been recently classified into proneural, neural, classical, and mesenchymal subtypes [6]. In comparison with other subtypes, mesenchymal-type GBM has been shown to be strongly associated with unfavorable outcomes, e.g., temozolomide (TMZ) resistance, in clinical patients. Since TMZ is a major chemotherapy drug used to treat GBM patients, uncovering the molecular mechanism underlying TMZ resistance is urgently needed.

Recent studies have demonstrated that 12% of GBM patients, especially young patients and patients with secondary GBM, acquire mutations in the active site of isocitrate dehydrogenase 1 (IDH1) [7,8]. IDH1 plays a substantial role in relieving cellular oxidative damage through the enhancement of NADPH production [7]. Prognostic estimations have revealed that an IDH1 mutation is associated with an increased survival probability in glioma patients who receive TMZ treatment [9,10]. In addition, the upregulation of O^6^-methylguanine DNA methyltransferase (MGMT), a DNA repair enzyme, has been associated with a poor response to TMZ treatment in GBM patients [11,12]. As a result, the hypermethylation of the GC island in the MGMT promoter has been used as a predictive marker for a prolonged survival time in GBM patients undergoing TMZ therapy [13]. Therefore, identifying more powerful biomarkers for predicting TMZ therapeutic effectiveness is worthwhile to more precisely estimate the outcomes of GBM patients who are deciding whether to receive TMZ treatment.

Histone 2A family members (H2AFs) are one of the components of nucleosomes and are crucial for gene regulation in the host [14]. Physiologically, H2AFX has been shown to promote the maintenance of genome integrity in male germ cells [15]. In breast cancer, copy number alterations and promoter genetic variations appear to correlate with breast carcinogenesis and the risk of sporadic breast cancer [16]. Accordingly, the genetic variation of the H2AFX promoter has been associated with the risk of glioma, particularly adult glioma [17]. On the other hand, the fusion of H2AFY with MDS1 and EVI1 Complex (MECOM) and the downregulation of H2AFZ have been associated with cancer progression in leukemia [18,19]. However, the clinical relevance of H2AFJ in cancers, including GBM, remains unknown.

Here, we show that H2AFJ is upregulated in primary tumors, compared to its expression in normal tissues derived from GBM patients. Moreover, our results indicate that the upregulation of H2AFJ, but not other H2AFs, is commonly found in mesenchymal-type GBM and is significantly associated with poor overall survival rates, probably due to the poor response to TMZ treatment in GBM patients with an unmethylated *MGMT* promoter region. Artificially silencing *H2AFJ* enhanced TMZ cytotoxicity against GBM cells, whereas overexpressing exogenous *H2AFJ* rendered GBM cells more resistant to TMZ treatment. Furthermore, we found that H2AFJ upregulation may be associated with the proneural-mesenchymal transition, which correlates with TMZ resistance [20] and likely activates TNF-α/NF-κB pathway which has been shown to mediate mesenchymal differentiation and therapeutic resistance in GBM cells [21]. Significantly, our results revealed that the therapeutic targeting of class I histone deacetylases (HDACs), e.g., HDAC3, by tacedinaline, which is a phase II clinical trial agent against advanced pancreatic cancer [22], might be a new strategy to combat TMZ-resistant GBM with H2AFJ upregulation.

## 2. Results

### 2.1. H2AFJ Is Frequently Upregulated in Mesenchymal-Type GBM Compared to Normal Brain Tissues and Low-Grade Gliomas

**Figure 1:**
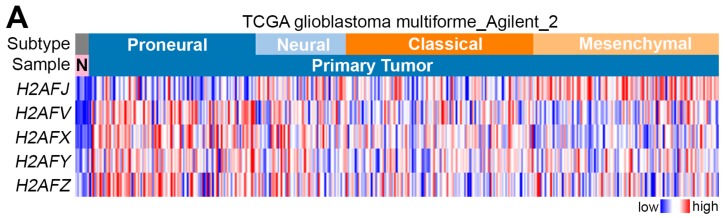

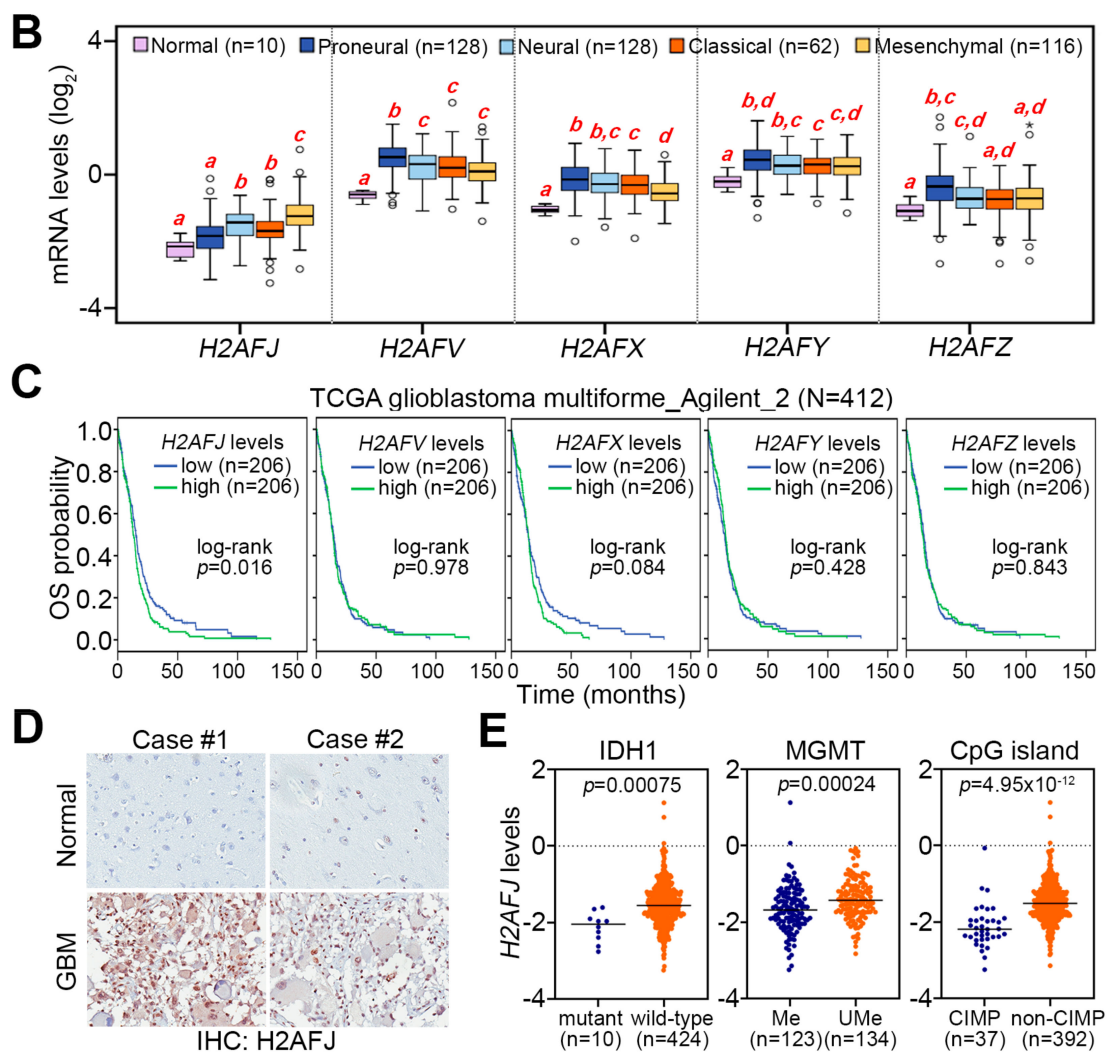
H2AFJ is highly expressed in mesenchymal-type GBM tissues. (**A**,**B**) Heatmap (**A**) and boxplot (**B**) for the transcriptional profile of the H2A subfamily, which was analyzed by Agilent G4502A microarray, in normal brain tissues (N for heatmap) and primary tumors derived from patients with different molecular subtypes (proneural, neural, classical and mesenchymal) of GBM using TCGA database. In (**B**), statistical significance was estimated by one-way ANOVA and Turkey’s post-hoc test. (**C**) Kaplan–Meier analyses for the mRNA levels of H2A subfamily under the condition of overall survival (OS) probability using TCGA GBM database. (**D**) Immunohistochemistry (IHC) staining of H2AFJ protein in two representatives of normal brain and GBM tissues. Photographs were taken at a magnification of 400×. (**E**) Dot plots for the transcriptional profiling of H2AFJ in IDH1 mutant and wild-type GBM, MGMT promoter methylated (Me), and unmethylated (Ume) GBM, or CpG island methylation phenotype (CIMP) and non-CIMP-harboring GBM. The statistical significance was determined by Student’s t-test.

We first analyzed the transcriptional profile of these genes analyzed by microarray method using Agilent_2 platform in TCGA normal brain tissues and GBM subtypes (pro-neural, neural, classical and mesenchymal) (Figure 1A). The results demonstrated that the mRNA levels of *H2AFJ*, *H2AFV*, *H2AFX*, *H2AFY*, and H2AFZ were upregulated in primary tumors compared to the levels in normal brain tissues derived from GBM patients (Figure 1B). Intriguingly, transcriptional profiling revealed that the gene expression of H2AFJ, but not other H2A histone family members (H2As), was significantly (*p* < 0.005) upregulated in mesenchymal-type GBM tissues but relatively lower in proneural-type GBM tissues (Figure 1A,B). In contrast, the transcripts of *H2AFV*, *H2AFX*, *H2AFY*, and *H2AFZ* were poorly expressed in mesenchymal-type GBM tissues but highly expressed in proneural-type GBM tissues (Figure 1A,B). Similar views were also observed in the dissection of their mRNA levels analyzed by RNA sequencing technique in TCGA normal brain tissues and GBM subtypes (Figure S1A,B). Kaplan–Meier analyses demonstrated that H2AFJ, but not other H2As, at higher mRNA levels determined by the median of its transcription profiling using Agilent microarray in TCGA GBM tissues significantly (*p* = 0.016) predict a poor overall survival probability (Figure 1C). Based on these findings, we thereafter focused on investigating the clinical relevance of H2AFJ in GBM.

Similar to the transcriptional levels, H2AFJ protein expression examined by immunohistochemistry staining was dramatically upregulated in GBM compared to normal brain tissues (Figure 1D) even though the sample size was not sufficient. Since IDH1 mutation, MGMT promoter methylation, and CpG island methylation phenotype (CIMP) have been widely used to estimate the effectiveness of radiation and TMZ therapies on GBM patients, we next analyzed the transcription profiling of H2AFJ in these molecular classifications. Whereas the mRNA levels of other H2As were not robustly different (Figure S2A–C), the mRNA levels of H2AFJ were significantly (*p* < 0.001) higher in GBM with wild-type IDH1, MGMT promoter unmethylation or non-CIMP, respectively, than in GBM with IDH1 mutations, MGMT promoter methylation or CIMP (Figure 1E). It suggests that H2AFJ upregulation might be associated with a poor radiation or TMZ response in GBM patients.

### 2.2. H2AFJ Upregulation Is Highly Correlated with a Poor Prognosis in Patients with Brain Tumors

**Figure 2:**
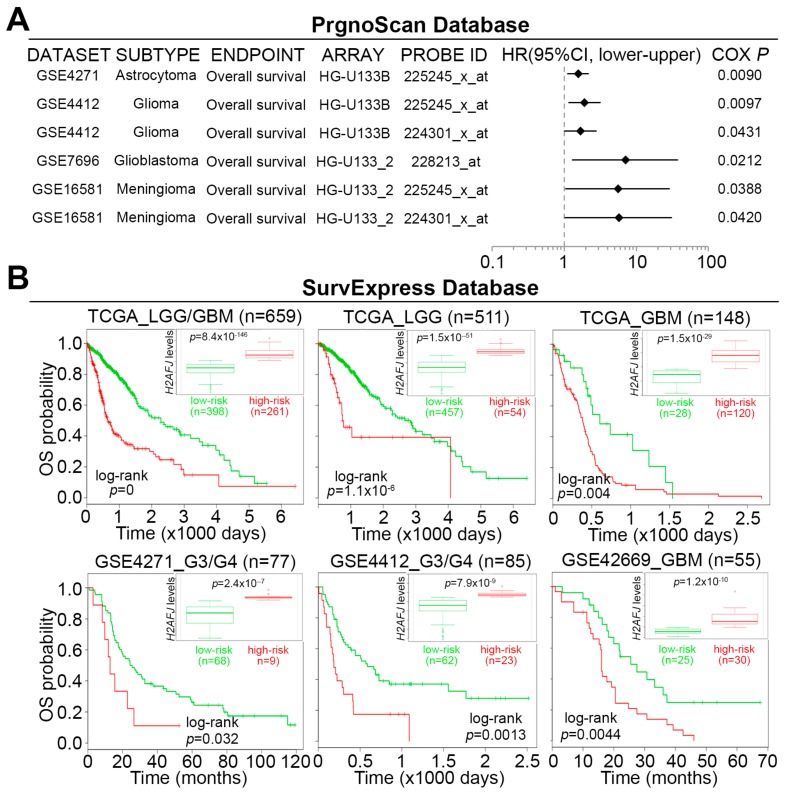

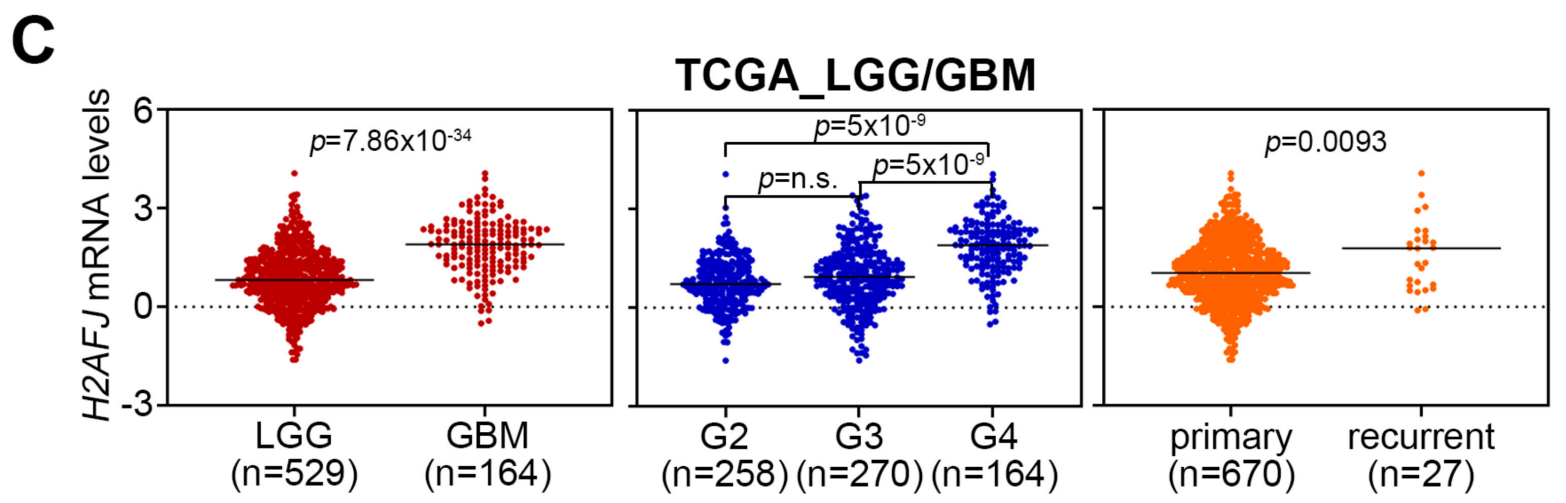
H2AFJ acts as a poor prognostic marker in brain tumors, particularly GBM. (**A**) A meta-analysis of *H2AFJ* in low-grade glioma and GBM using the PrognoScan database (left). A forest plot (**right**) was used to present the hazard ratio (HR) distribution in the 95% confidence interval (CI) estimated by Cox regression against H2AFJ transcript levels (high vs. low) in the enrolled cohorts. (**B**) Prognostic estimation of H2AFJ expression using the SurvExpress program under the condition of overall survival (OS) probability in low-grade glioma (LGG)/GBM, LGG or GBM cohorts from TCGA and LGG or GBM cohorts from three GEO datasets (GSE4271, GSE4412, and GSE42669). The inserts indicate the mRNA levels of H2AFJ in the low- and high-risk groups. (**C**) Dot plots for the transcriptional profiling of H2AFJ in clinical tissues derived from LGG and GBM, different histological grades, or primary and recurrent tumors using TCGA LGG/GBM database. The bars indicate the mean of H2AFJ mRNA levels in each group. The statistical significance was analyzed by Student t-test (left and right) or one-way ANOVA and Turkey’s post-hoc test (middle). The symbol “n.s.” denotes not significant.

To understand the clinical relevance of *H2AFJ*, we next performed a meta-analysis using the PrognoScan database (http://www.prognoscan.org/). Our data showed that *H2AFJ* upregulation was associated with unfavorable hazard ratios in different types of cancer, especially in brain tumors (Figure 2A). We further validated these findings by performing a Kaplan–Meier analysis for patients with low-grade glioma (LGG) and/or GBM from TCGA using the SurvExpress program [23]. The data showed that an elevated level of H2AFJ transcript predicts a poor overall survival (OS) rate in the unclassified patients or patients with LGG or GBM in a Kaplan–Meier analysis under a maximal risk condition (Figure 2B). To further confirm the prognostic significance of H2AFJ in GBM, we utilized three Gene Expression Omnibus (GEO) datasets GSE13041 [24], GSE4412 [25], and GSE42669 [26] to perform another Kaplan–Meier analysis of H2AFJ gene expression. The data showed that the elevation of the H2AFJ transcript was significantly (*p* < 0.01) associated with a poor survival rate in glioma patients in those three datasets (Figure 2B). Furthermore, the transcriptional profiling revealed that the expression of H2AFJ in GBM, grade IV glioma and recurrent tumors is more abundant than that in LGG, grade II/III glioma and primary tumors, respectively, in TCGA LGG/GBM database (Figure 2C). Whereas the upregulation of H2AFJ was evident for brain tumors from patients with age equal to or over 64 years, its mRNA levels appeared to be no different in brain tumors from female and male patients (Figure S3A,B). Again, these findings indicated that H2AFJ upregulation may refer to a poor prognosis and associate with a poor therapeutic response in brain tumors.

### 2.3. H2AFJ Expression Determines TMZ, not Radiation, Effectiveness in GBM.

**Figure 3:**
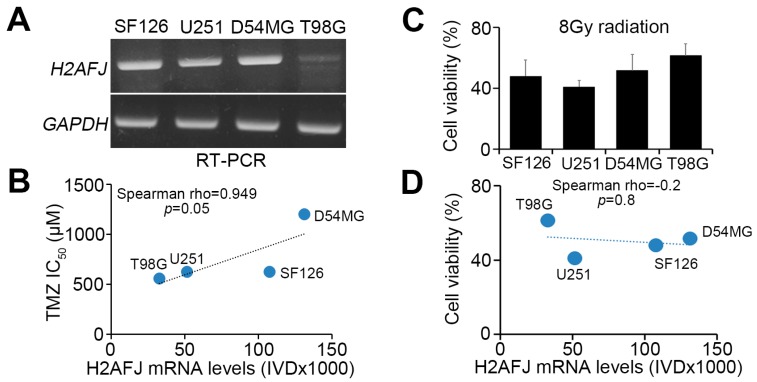

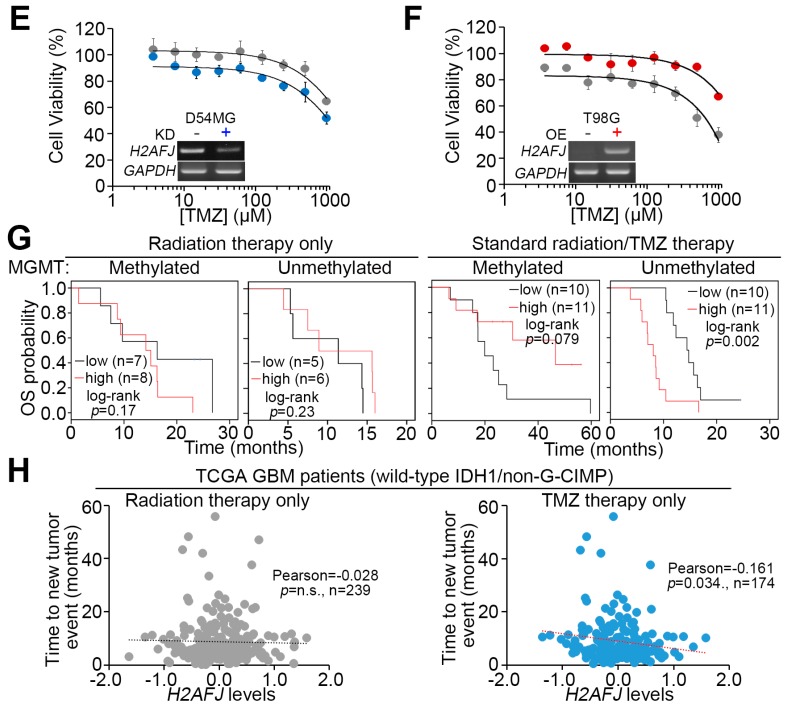
H2AFJ upregulation desensitizes GBM cells to TMZ treatment. (**A**) RT-PCR analysis of *H2AFJ* and *GAPDH* expression in different GBM cell lines. (**B**) Scatter plots for the correlation between H2AFJ expression and TMZ IC_50_ concentrations in various GBM cells. (**C**) Cell viability of the detected GBM cells after 24-h exposure to radiation at 8 Gy. The count of remaining viable cells after radiation treatment was normalized with the cell number of the untreated group in each detected GBM cell line. The data from three independent experiments are presented as the mean ± SEM. (**D**) Scatter plots for the correlation between H2AFJ mRNA levels and cell viability of the detected GBM cells posttreatment with radiation at 8 Gy for 24 h. A non-parametric Spearman correlation test was used to estimate statistical significance in **B** and **D**. (**E**,**F**) Scatchart plots for the cell viability of D54MG cells (**E**) with or without *H2AFJ* knockdown (insert) and T98G cells (**F**) with or without *H2AFJ* overexpression (insert) after TMZ treatment for four days at the indicated TMZ concentrations. In **A**, **E** and **F**, GAPDH was used as an internal control for RT-PCR experiments. (**G**) Kaplan–Meier analysis of overall survival probability associated with H2AFJ gene expression in MGMT-unmethylated and -methylated GBM patients undergoing radiation therapy or standard TMZ/radiation therapy. (**H**) Scatter plots for H2AFJ transcripts and time to a new tumor event after radiation or TMZ therapy in GBM patients who were diagnosed to be wild-type IDH and non-G-CIMP. The statistical significance was estimated by Pearson’s correlation test. The symbol “n.s.” denotes not significant.

To ascertain the association of H2AFJ expression with the therapeutic effectiveness of radiation and TMZ on GBM, we next validated this phenomenon in GBM cell lines. We found that the endogenous mRNA levels of *H2AFJ* and *MGMT* were causally associated (Figure 3A) and significantly (*p* = 0.05) correlated with the 50% inhibitory concentration (IC_50_) of TMZ (Figure 3B) but not the cytotoxic effectiveness of radiation (Figure 3C,D) in the analyzed GBM cell lines. Whereas *H2AFJ* knockdown sensitized D54MG glioma cells to TMZ treatment (Figure 3E), *H2AFJ* overexpression rendered T98G glioma cells more resistant to TMZ treatment (Figure 3F).

Since the methylation of the MGMT promoter has been correlated with a favorable TMZ response, we next examined the prognostic significance of H2AFJ in patients receiving radiation therapy combined with or without TMZ treatment and in GBM with or without MGMT methylation using the GSE7696 dataset. In patients receiving radiation therapy (RT) only, regardless of MGMT methylation status, H2AFJ gene expression was not significantly correlated with an overall survival probability of he enrolled GBM patients in a Kaplan–Meier analysis (Figure 3G). In patients receiving combined treatment of radiation and TMZ followed by TMZ therapy only, *H2AFJ* upregulation significantly (*p* < 0.01) predicted a poor overall survival rate in patients with MGMT-unmethylated GBM (Figure 3G). In contrast, in patients with MGMT-methylated GBM, *H2AFJ* expression was causally associated with overall survival time after treatment with radiation and TMZ therapy (Figure 3G). Similarly, in TCGA cohorts with IDH1 wild-type GBM without glioma CpG island methylation phenotype (G-CIMP), the increased mRNA levels of H2AFJ was significantly (*p* = 0.034) correlated with a shorter time to new tumor event after patients receiving TMZ, not radiation, therapy (Figure 3H). Therefore, H2AFJ may serve a predictive biomarker for the TMZ effectiveness in GBM.

### 2.4. H2AFJ Upregulation Associates with the Progression of Proneural-Mesenchymal Transition and the Activation of TNFα-NF-κB and IL6-STAT3 Signaling Pathways in GBM

**Figure 4:**
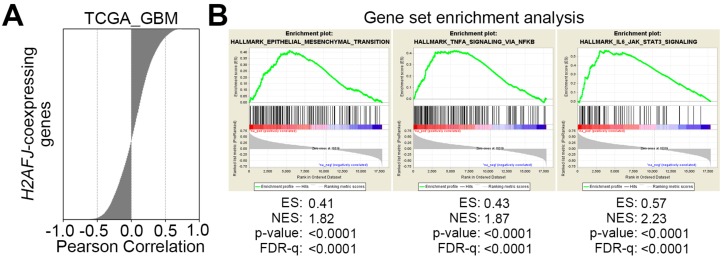

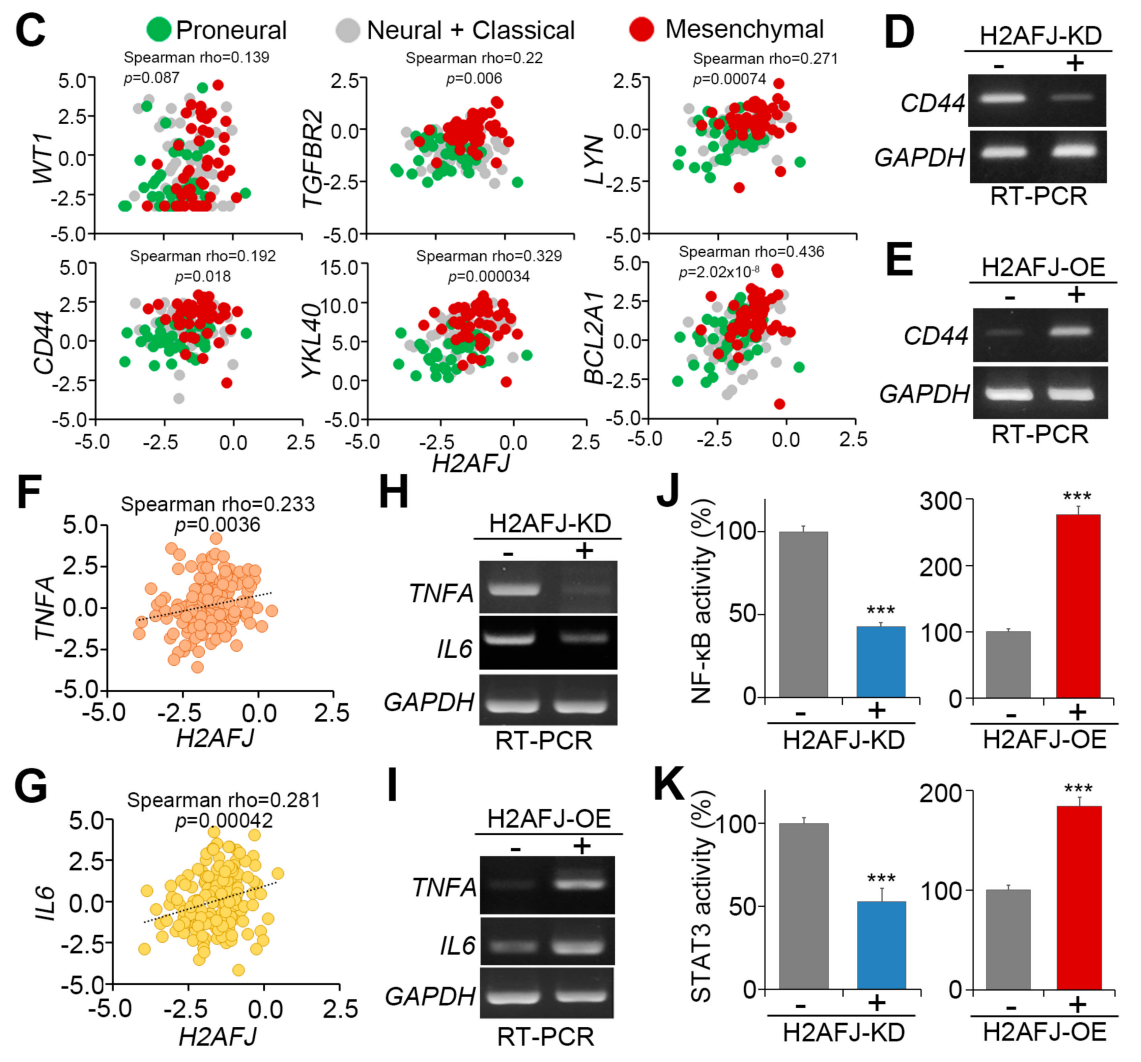
H2AFJ upregulation is associated with proneural-mesenchymal transition and activation of TNFα/NF-κB and IL-6/STAT3-related pathways. (**A**) Histogram for the results from Pearson’s correlation test of the transcriptional profile for H2AFJ and other somatic genes in TCGA GBM tissues. (**B**) Snapshot of gene set enrichment analysis (GSEA) results showing as enrichment plots for epithelial-mesenchymal transition, TNFα/NF-κB and IL-6/STAT3 gene sets using the H2AFJ-related gene signature in **A**. (**C**) Scatter plots for the correlation of transcriptional profiles among *H2AFJ* and proneural-mesenchymal transition-associated markers (*WT1*, *TGFBR2*, *LYN*, *CD44*, *YKL40*, and *BCL2A1*) in GBM tissues from TCGA. (**D**,**E**) RT-PCR analysis for the mRNA levels of *CD44* and *GAPDH* in D54MG cells without or with *H2AFJ* knockdown (KD, **D**) and T98G cells without or with *H2AFJ* overexpression (OE, **E**). (**F**,**G**) Scatter plots for the correlation among the gene expression of TNF-alpha (TNFA, **F**), interleukin-6 (IL6, **G**) and H2AFJ in the TCGA GBM database. In **C**, **F**, **G**, the statistical significance was analyzed by Spearman’s correlation test. (**H**,**I**) RT-PCR analysis for the mRNA levels of *TNFA*, *IL6,* and *GAPDH* in D54MG cells without or with *H2AFJ* knockdown (KD, **H**) and T98G cells without or with *H2AFJ* overexpression (OE, **I**). In **D**, **E**, **H** and **I**, *GAPDH* was used as an internal control for RT-PCR. (**J**,**K**) Histograms represent the results of luciferase-based reporter assays for NF-κB (**J**) and STAT3 (**K**) activities in D54MG cells without (non-silencing control, NS) or with *H2AFJ* knockdown (KD) and T98G cells without (vector control, VC) or with H2AFJ overexpression (OE). The data from three independent experiments are presented as the mean ± SEM. “***” denotes statistical significance at *p* < 0.001 for a non-parametric Mann–Whitney test.

To realize the possible mechanism for H2AFJ-promoted TMZ resistance in GBM, we next performed an in silico analysis. We performed a Pearson correlation test for the co-expression of H2AFJ gene with other somatic genes in TCGA GBM tissues (Figure 4A) from cBioPortal. We next analyzed this gene signature by Gene Set Enrichment Analysis (GSEA). The GSEA results showed that this H2AFJ-related gene signature was significantly (*p* < 0.001) correlated with the upregulation of epithelial-mesenchymal transition (EMT), TNF-alpha/NF-κB and IL-6/STAT3 pathways (Figure 4B). Because the induction of proneural-mesenchymal transition (PMT) has been shown to be similar to EMT and correlated with a mechanism for TMZ resistance in GBM cells [20], we next analyzed the correlation between the expression of the H2AFJ gene and PMT signature genes, which include *WT1*, *TGFBR2*, *LYN*, *CD44*, *YKL40*, and *BCL2A1*, in the GBM tissues from TCGA. The data revealed that H2AFJ gene expression was highly correlated with the mRNA levels of PMT signature genes (Figure 4C). Whereas *H2AFJ* knockdown in D54MG cells repressed the gene expression of CD44 (Figure 4D), *H2AFJ* overexpression in T98G cells enhanced the transcription of CD44 (Figure 4E). In addition, the mRNA levels of TNF-alpha and H2AFJ (Figure 4F), as well as interleukin-6 and H2AFJ (Figure 4G), were shown to be positively correlated in the TCGA GBM database. Artificially silencing the expression of *H2AFJ* predominantly repressed the mRNA levels of TNF-alpha and interleukin-6 in D54MG glioblastoma cells (Figure 4H), whereas the forced gene expression of ectopic *H2AFJ* dramatically enhanced the expression of TNF-alpha and interleukin-6 in T98G glioblastoma cells (Figure 4I). Moreover, a luciferase-based reporter assay demonstrated that *H2AFJ* knockdown reduced but overexpression elevated the transcription factor activities of NF-κB (Figure 4J) and STAT3 (Figure 4K) in the tested D54MG and T98G cells, respectively.

**Figure 5:**
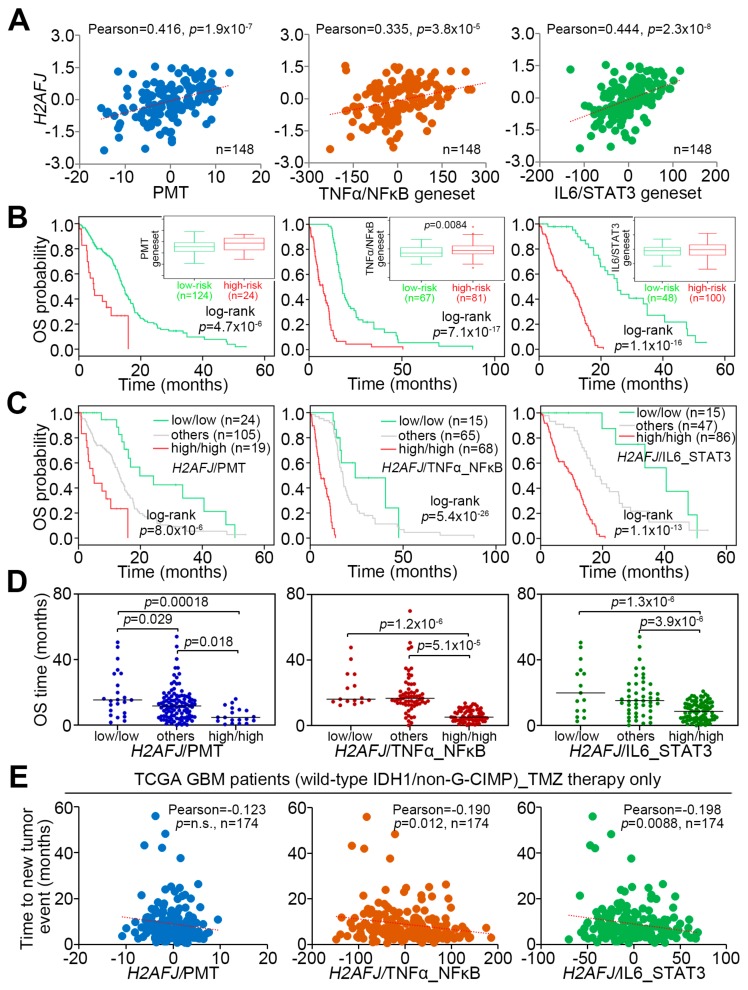
The signature of combing high-level H2AFJ transcripts with high-level PMT geneset, TNFα/NF-κB geneset or IL-6/STAT3 geneset expression correlates with poor responsiveness to TMZ therapy in patients with GBM harboring wild-type *IDH1* and non-G-CIMP. (**A**) Scatter plots for the correlation of mRNA levels determined by RNA sequencing technique among *H2AFJ*, PMT geneset, TNFα/NF-κB geneset or IL-6/STAT3 geneset in primary tumors derived from the TCGA GBM database. (**B**,**C**) Kaplan–Meier analyses using overall survival (OS) probability for the low and high-risk populations defined by SurvExpress program under a maximal risk condition in accordance with the transcriptional levels of PMT geneset, TNFα/NF-κB geneset or IL-6/STAT3 geneset combined without (**B**) or with (**C**) H2AFJ mRNA levels in TCGA GBM database. Insets represent the mRNA levels of the factors in low and high-risk populations. In **C**, others denote the low/high and high/low expression levels. (**D**) Dot plots represent overall survival time in the three populations stratified in **C** according to the transcriptional profiles of H2AFJ, PMT geneset, TNFα/NF-κB geneset or IL-6/STAT3 geneset in primary tumors derived from the TCGA GBM database. The bars indicate the mean of overall survival time in each group. One-way ANOVA and Turkey’s post-hoc test was used to estimate the statistical significance. (**E**) Scatter plots for the correlation of time to new tumor event with the H2AFJ mRNA levels combined with the transcriptional profiles of PMT geneset, TNFα/NF-κB geneset or IL-6/STAT3 geneset in the TCGA IDH1 wild-type/non-G-CIPM GBM patients receiving TMZ therapy. The statistical significance was analyzed by Pearson’s correlation test. The symbol “n.s.” denotes not significant.

By using the TCGA database, we found a significantly (*p* < 0.0001) positive correlation among the mRNA levels of H2AFJ, PMT geneset, TNFα-NF-κB geneset, and IL6-STAT3 geneset in GBM tissues (Figure 5A). Moreover, Kaplan–Meier analyses under a maximal risk condition demonstrated that high-level expression of PMT, TNFα-NF-κB, and IL6-STAT3 genesets refers to a poor overall survival probability TCGA GBM patients (Figure 5B). Robustly, compared to the combination of low-level H2AFJ with low-level expression of PMT, TNFα-NF-κB, or IL6-STAT3 geneset, as well as other combinations, the scenario of combining high-level of *H2AFJ* with high-level expression of PMT, TNFα-NF-κB, or IL6-STAT3 geneset predicted a worse prognosis (Figure 5C) and shorter overall survival time (Figure 5D) in TCGA GBM patients. In TMZ-receiving patients with GBM harboring wild-type *IDH1* and non-G-CIMP, the combined expression of *H2AFJ* with PMT, TNFα-NF-κB, or IL6-STAT3 geneset exhibited an inverse correlation with time to new tumor event after the treatment (Figure 5E). It might indicate that the upregulation of *H2AFJ* accompanied by the activation of PMT, TNFα-NF-κB, and IL6-STAT3 pathways is associated with the mechanism for TMZ resistance in GBM.

### 2.5. Pharmaceutical Inhibition of NF-κB and HDAC3 Provides a New Strategy for Overcoming TMZ-Insensitive GBM with H2AFJ Upregulation

**Figure 6:**
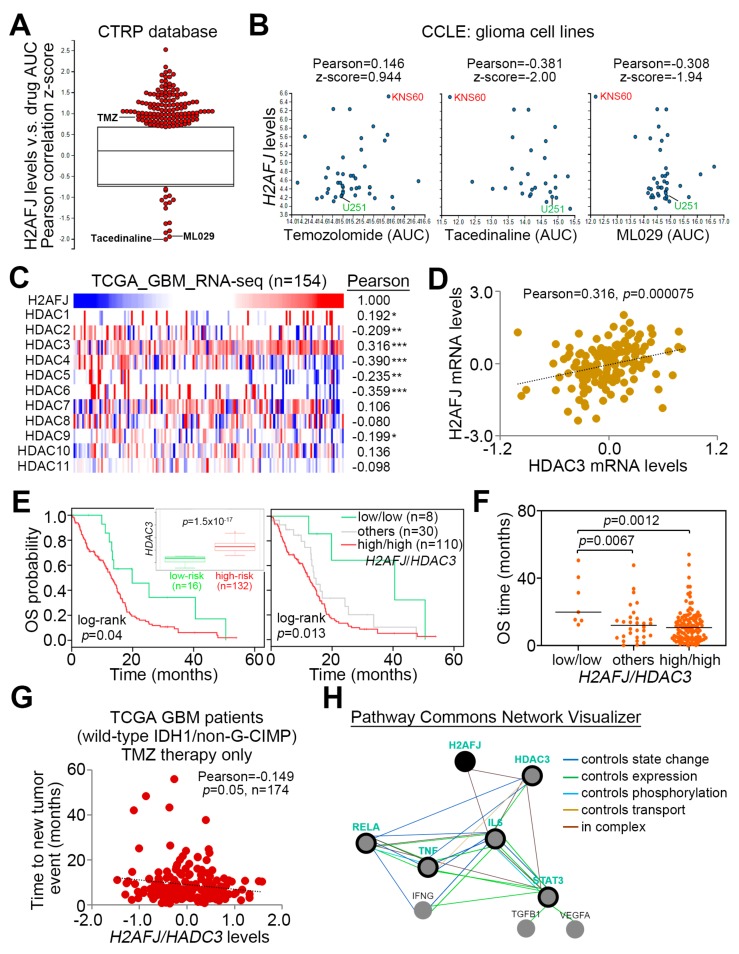
(**A**) Box/dot plot for the z-score derived from Pearson’s correlation test for the transcriptional profile of H2AFJ and the AUC values of drug IC_50_ concentrations in glioma cell lines using CTRP database. AUC is abbreviated from the area under the curve of the receiver operating characteristic curve. (**B**) Scatter plots represent the results of Pearson’s correlation test for the association of H2AFJ mRNA levels with the AUC values of temozolomide, tacedinaline or ML029 IC_50_ concentrations in glioma cell lines using the Cancer Cell Line Encyclopedia (CCLE) database. (**C**) Heatmap for the transcriptional profiles of H2AFJ and HDAC subfamily determined by RNA sequencing method in the TCGA GBM database. The results of Pearson’s correlation test against the transcriptional profiles of H2AFJ and the all listed genes are shown next to the heatmap. The symbols *, ** and *** denote the statistical significance at *p* < 0.05, *p* < 0.01, and *p* < 0.001 in Pearson’s correlation test. (**D**) Scatter plot represents the correlation for the transcriptional profile of H2AFJ and HDAC3 in the TCGA GBM database. (**E**) Kaplan–Meier analyses using overall survival (OS) probability for the low and high-risk populations defined by SurvExpress program under a maximal risk condition in accordance with the transcriptional profile of HDAC3 combined without (left) or with (right) H2AFJ mRNA levels in TCGA GBM database. Insets represent the mRNA levels of HDCA3 in low and high-risk populations. In the right figure, others denote the low/high and high/low expression levels of *H2AFJ* and *HDAC3*. (**F**) Dot plots represent overall survival time in the three population stratified in E according to the transcriptional profiles of H2AFJ and HDCA3 in primary tumors derived from the TCGA GBM database. The bars indicate the mean of overall survival time in each group. One-way ANOVA and Turkey’s post-hoc test was used to estimate the statistical significance. (**G**) Scatter plots for the correlation of time to a new tumor event with the transcriptional profile of H2AFJ/HDAC3 in TCGA IDH1 wild-type/non-G-CIPM GBM patients receiving TMZ therapy. The statistical significance was analyzed by Pearson’s correlation test. (**H**) The network of inter-molecular interactions among H2AFJ, HDAC3, TNFα/NF-κB (RELA), and IL-6/STAT3 using Pathway Commons Network Visualizer program. The relationships of inter-molecular interaction are shown in the inserted panel.

To combat the TMZ resistance due to *H2AFJ* upregulation in GBM, we next performed an in silico genomic-based drug discovery using the Cancer Therapeutics Response Portal (CTRP) database [27,28]. The Pearson’s correlation test against H2AFJ mRNA levels and the IC_50_ AUC values of deposited compounds showed that the IC_50_ concentrations of tacedinaline, a selective inhibitor for class I histone deacetylase (HDAC) 1, 2, 3, and 8, and ML029, an inhibitor for NF-κB negatively correlates with H2AFJ mRNA levels in a panel of glioma cell lines (Figure 6A,B and Figure S4). In contrast, the IC_50_ concentrations of TMZ appeared to be positively correlated with H2AFJ transcriptional profile in the detected glioma cells (Figure 6A,B and Figure S4). The negative correlation of H2AFJ expression with tacedinaline or ML029 IC_50_ concentrations but its positive correlation with TMZ IC_50_ concentrations was evident for KNS60 and U251 glioma cells (Figure 6B and Figure S4). Furthermore, we performed transcriptional profiling of HDAC family in order to identify a highly co-expressing HDAC with *H2AFJ* in GBM (Figure 6C). We found that the expression of *H2AFJ* and *HDAC3* is significantly (*p* < 0.001) and positively correlated in the primary tumors of TCGA GBM database (Figure 6D). While *HDAC3* upregulation was correlated with a poor prognosis, the scenario of combining high-level expression of *H2AFJ* and *HDAC3* predicted a worse overall survival probability in the TCGA GBM patients in Kaplan–Meier analyses using a maximal risk condition (Figure 6E). Accordingly, in comparison with other combinations, the combination of high-level *H2AFJ* and *HDAC3* expressions was significantly (*p* < 0.01) associated with a shorter overall survival time of GBM patients (Figure 6F). Moreover, the combined expression of *H2AFJ* and *HDAC3* was appeared to be inversely correlated with the time to new tumor event after TMZ therapy in the wild-type IDH1/non-G-CIMP GBM patients (Figure 6G). Finally, the computational simulation using Pathway Commons Network Visualizer revealed a putatively inter-molecular interaction among H2AFJ with HDAC3, TNFα/NF-κB, and IL6/STAT3 (Figure 6H). These findings may provide tacedinaline or ML029 as an alternative strategy to treat TMZ-insensitive GBM with *H2AFJ* upregulation.

## 3. Discussion

Based on gene expression patterns and clinical characteristics, GBM has been classified into four subtypes: Proneural, neural, classical, and mesenchymal [6]. Among these subtypes, mesenchymal-type GBM exhibits the most aggressive phenotypes (e.g., TMZ resistance), and therefore, results in a worse prognosis in GBM patients [6,29]. Here, we show that H2AFJ differs from other H2A subfamily members in that it is highly expressed by mesenchymal-type GBM tissues and serves as a predictive factor for TMZ effectiveness in GBM patients. Moreover, artificially silencing *H2AFJ* sensitized but overexpressing *H2AFJ* desensitized GBM cells to the cancericidal effect of TMZ. In GBM patients who lack methylation at the *MGMT* promoter region, a GBM subpopulation presumably insensitive to TMZ treatment [30,31], we found that *H2AFJ* upregulation is able to further differentiate a poorer TMZ-responsive subgroup from those patients. In addition, our data showed that *H2AFJ* upregulation is extensively detected in GBM tissues with wild-type *IDH1*, another indicator for a poor TMZ response [10,12]. Notably, a meta-analysis revealed that *H2AFJ* upregulation is a poor prognostic marker in brain tumors, irrespective of pathological grade. Based on these findings, we propose that the combination of *H2AFJ* levels with *MGMT* and/or *IDH1* features is likely able to more precisely identify brain tumor patients who will have good outcomes after TMZ therapy.

Proneural-mesenchymal transition (PMT) has been identified as a process of cancer progression and is related to the mechanism for TMZ resistance in GBM [20]. The induction of PMT post-radiation therapy through the activation of the NF-κB and STAT3 pathways by inflammatory agents, such as IL-6, TNF-α, etc., has been found in GBM [32,33,34]. Moreover, IL-6 overexpression has been identified as a marker of malignancy in GBM [35]. Here, we showed that *H2AFJ* expression was causally associated with the PMT process, as judged by a positive correlation between *H2AFJ* and PMT signature gene mRNA levels in GBM tissues. Moreover, we found that *H2AFJ* knockdown dramatically suppressed IL-6 expression and NF-κB activity in GBM cells. Although a more comprehensive mechanism by which H2AFJ regulates the IL-6-NF-κB axis to modulate PMT progression should be further explored in GBM, our findings may be the first to document that the upregulation of *H2AFJ*, but not of other H2A subfamily members, is associated with the molecular mechanism for PMT progression, which confers TMZ resistance in GBM.

NF-κB has been identified as a transcriptional regulator of MGMT [31,36]. The overexpression of ectopic p65 or high constitutive NF-κB activity has been shown to elevate MGMT and thereby enhance chemoresistance to alkylating agents in detected cells [37]. In contrast, the inhibition of NF-κB by the small molecule inhibitor BAY 11-7082 or by siRNA-mediated gene silencing reduced *MGMT* levels and thereby sensitized glioma stem-like cells to TMZ treatment [30,32]. Here, we found that *H2AFJ* expression causally modulates the activity of NF-κB in GBM cells. Based on these findings, we hypothesize that H2AFJ acts as an upstream regulator of NF-κB-mediated *MGMT* expression and PMT induction, which play crucial roles in conferring TMZ resistance in GBM. Moreover, an in silico simulation using Pathway Commons Network Visualizer program demonstrated that the interaction of HDAC3 with H2AFJ may epigenetically regulate the elevation of the IL-6 transcription, thereby enhancing the activity of IL-6/STAT3 signaling axis which subsequently triggers the activation of TNF-α/NF-κB pathway via elevating the gene expression of TNF-α. Although our results revealed that the gene expression of IL-6 and TNF-α is causally affected after *H2AFJ* knockdown and overexpression in the detected cells, further experiments are still needed to clarify the role of HADC3 in regulating H2AFJ activity by a post-translational modification of lysine acetylation and identify if the genomic DNA of IL-6, as well as other related molecules, is recruited as the component of H2AFJ-associated nucleosome in GBM.

Post-translational modifications (PTMs) of histones by the epigenetic enzymes, e.g., HDACs, is essential for their protein functions in regulating gene expression during normal cell development [38,39] and cancer progression [40,41]. Since histone variants are highly conserved between different species, their functions cannot be accomplished by canonical histones [42]. The altered expression of H2As has been found in cancers and resulted in different consequences. Whereas the H2AFX and H2AFY are considered as tumor suppressors, H2AFZ exhibits an oncogenic function [43]. Here, we further show that H2AFJ likely serves as a driver in promoting TMZ resistance and may be associated with the mesenchymal differentiation in GBM. Therefore, targeting H2AFJ functions could be a new strategy to overcome the most aggressive mesenchymal-GBM. Nevertheless, the use of additional genetic brain tumor profiling resources (e.g., longitudinal tumor trajectories [44]) and further experimental validation in diverse genotypic context *(IDH1* mutant cell models, etc.) is still needed to clarify whether or how the phenotype of H2AFJ upregulation is linked to a specific clonal or subclonal evolution of a clinically-relevant genotype of brain tumor progression and therapeutic resistance. In addition, further in vitro and in vivo experiments (i.e., utilizing a spectrum of patient-derived models and tumor stem cell contexts) are also required for validating the potential therapeutic significance of elevated H2AFJ in glioblastoma resistance working toward patient stratification.

## 4. Materials and Methods

### 4.1. Clinical and Molecular Data for Patients

Raw data for the pathologic information and prognostic value of *H2AFJ* were downloaded from the PrognoScan database (http://www.abren.net/PrognoScan/). Datasets with a statistical *p*-value < 0.05 in a Cox regression analysis were included in a meta-analysis for H2AFJ gene expression. Transcriptional profiling of H2AFs was obtained from The Cancer Genome Atlas (TCGA, data released in Jan 2016) and Gene Expression Omnibus (GEO) databases and subjected to statistical analysis for the differential display of their mRNA in subgroups. A Kaplan–Meier analysis and the differential display for H2AFJ gene expression were performed on the SurvExpress website or using SPSS software.

### 4.2. Cell Lines and Cell Culture Conditions

GBM cell lines obtained from the American Type Culture Collection (ATCC, Manassas, VA, USA) were maintained in conditioned media. We maintained 293T cells in DMEM supplemented with 10% fetal bovine serum (FBS). Cells were incubated at 37 °C with 5% CO_2_. All cells were routinely authenticated on the basis of short tandem repeat (STR) analysis, morphologic and growth characteristics and mycoplasma detection.

### 4.3. MTT Assay

Cells (5 × 10^4^/mL) were seeded into a 96-well culture plate. After incubation, 10 μL of MTT (3-(4,5-dimethylthiazol-2-yl)-2,5-diphenyltetrazolium bromide, molecular probe, (Invitrogen, Carlsbad, CA, USA) stock solution was added to each well. The conversion of MTT to formazan by viable cells was performed at 37 °C for 4 h. After the reaction, 100 μL of DMSO solution was added to each well to solubilize the formazan precipitates. The levels of formazan were determined by optical density at 540 nm using an ELISA reader for calculating cell survival rates.

### 4.4. Plasmid Construction

The gene encoding H2AFJ was amplified from human cDNA (Invitrogen) using a standard polymerase chain reaction (PCR) procedure with paired primers and subcloned into the pDONR221 vector. The identities of individual clones were verified via double-strand plasmid sequencing. The pDONR221 plasmid was recombined with the destination vector pLenti6.3/V5-DEST in 293T cells to create packaged lentiviral particles. A commercially available plasmid, pLenti/GFP, sharing the same backbone (Invitrogen) was used as a control. The recombinant lentiviruses in the culture medium were harvested, concentrated using a Lenti-X Concentrator (Clontech, Mountain View, CA, USA), and then titrated by determining the viral RNA genome content with a Lenti-X qRT-PCR titration kit (Clontech) according to the manufacturer’s instructions.

### 4.5. Lentivirus-Driven shRNA Infection

Lentiviral shRNA constructs were purchased from Open Biosystems. Lentiviruses were produced by cotransfecting the shRNA-expressing vector with the pMDG and p△8.91 constructs into 293T cells using a calcium phosphate transfection kit (Invitrogen). After incubation for 48–72 h, the media were collected as viral stocks. Cells (50% confluence) grown on six-well plates were incubated in fresh media containing 5 μg/mL polybrene (Santa Cruz Biotechnology, Santa Cruz, CA, USA) before infection overnight with a lentiviral viral particle-driven control or candidate gene shRNA at 2–10 multiplicity of infection (MOI). To select cells stably expressing the control or candidate gene shRNA, cells were further cultivated in the presence of puromycin (10 μg/mL) for 24 h. Cell lysates from the puromycin-resistant cells were subsequently subjected to PT-RCR analysis to confirm the efficiency of gene knockdown.

### 4.6. Reverse Transcription PCR (RT-PCR)

Total RNA was extracted from cells using a TRIzol extraction kit (Invitrogen). Aliquots (5 μg) of total RNA were treated with M-MLV reverse transcriptase (Invitrogen) and then amplified with Taq-polymerase (Protech) using paired primers (for *H2AFJ*, forward-CTGGATGTTGGGCAGGACG and reverse-GAAACAGGGCGGCAAAGTGC, for *MGMT*, forward-TCATCCCGTGCCACAGA and reverse-AGCGGTGCCTCCACG, for *TNF*, forward-TGGCCCAGGCAGTCAGAT and reverse-GCAGCCTTGGCCCTTGAA, for *IL6*, forward-CTGGATGTTGGGCAGGACG and reverse-GAAACAGGGCGGCAAAGTGC, and for *GAPDH*, forward-AGGTCGGAGTCAACGGATTTG and reverse-GTGATGGCATGGACTGTGGTC).

### 4.7. Irradiation Treatment and Cell Viability Analysis

Irradiation was performed with 6 MV X-rays using a linear accelerator (Digital M Mevatron Accelerator, Siemens Medical Systems, Ann Arbor, MI, USA) at a dose rate of 8 Gy/min. An additional 2 cm of a tissue-equivalent bolus was placed on the top of the plastic tissue-culture flasks to ensure electronic equilibrium, and 10 cm of tissue-equivalent material was placed under the flasks to obtain full backscatter. After IR treatment, cells were centrifuged and resuspended with an appropriate amount of PBS. For the cell viability assay, 20 μL of the cell suspension was mixed with 20 μL of Trypan blue solution (0.4% in PBS). The stained cells were placed on a hemocytometer, and the blue-stained cells were counted as nonviable under a microscope.

### 4.8. Luciferase Reporter Assay

Luciferase reporter vectors containing NF-κB or sis-inducible element (SIE) response elements within the promoter region were purchased from Promega and utilized to estimate the activity of NF-κB or STAT3, respectively. Cells were seeded in six-well plates and co-transfected with a firefly luciferase reporter and renilla-expressing vectors. After 24 h, luciferase activity was measured using a Dual-Glo^®^ Luciferase Assay System (Promega, Madison, WI, USA). In brief, the cells were lysed in lysis buffer containing luciferase substrate for 10 min. Total lysis was achieved with centrifugation at 12,000 rpm for 1 min, and the supernatant was split into three wells in a white 96-well plate to measure firefly luminescence. Dual-Glo^®^ Stop & Glo^®^ reagent was added to each well. After 10 min, renilla luminescence was measured. The level of firefly luminescence was normalized to renilla luminescence.

### 4.9. Immunohistochemistry Staining Analysis

Paraffin-embedded tumor sections (3 μm thick) were heated, deparaffinized using xylene, and rehydrated in a graded series of ethanol with a final wash in tap water. Antigen retrieval was performed with target retrieval solution (DAKO) in a decloaking chamber (Biocare Medical, Pacheco, CA, USA). Endogenous peroxidase activity was quenched with hydrogen peroxide. The sections were then incubated with anti-H2AFJ antibody (Sigma-Aldrich, HPA041189, St. Louis, MO, USA) at 4 °C overnight. A VECTASTAIN ABC peroxidase system (Vector Laboratories, Burlingame, CA, USA) was used to detect the reaction products. Commercially available tissue microarrays were produced by US Biomax Inc. (Rockville, MD, USA) and all human tissues were collected under the Health Insurance Portability and Accountability Act (HIPPA, U.S. Government Publishing Office) approved protocols. Detailed information on all tumor specimens can be found at http://www.biomax.us/index.php. The study was approved by Cardinal Tien Hospital institutional review board approval (CTH-101-3-5-054) in accordance with the Declaration of Helsinki.

### 4.10. Statistical Analysis

SPSS 17.0 software (Informer Technologies, Chicago, IL, USA) was used to analyze statistical significance. One-way ANOVA and Tukey’s test were utilized to compare the differential display of H2AFJ gene expression in normal tissues and primary tumors. Pearson’s and Spearman tests were performed to estimate the association between H2AFJ levels and the detected parameters. Evaluation of the survival probabilities was determined by Kaplan–Meier analysis and the log-rank test. The Mann–Whitney test was used to analyze the nonparametric data. A *p*-value < 0.05 in all analyses was considered to be statistically significant.

## 5. Conclusions

This study is the first to document that a histone 2A subfamily member, H2AFJ, has an oncogenic function and is highly expressed in primary tumors of GBM, especially mesenchymal-type GBM, which commonly exhibits a poor TMZ response and unfavorable outcomes in patients. Our results demonstrate that the upregulated expression of *H2AFJ* is likely associated with the activation of TNF-α−NF-κB/IL-6-STAT3 signaling pathways and the recruitment of class I HDAC activity to promote PMT and TMZ resistance in GBM. Fortunately, the finding that HDAC inhibitor tacedinaline, currently a phase II anti-cancer agent, is capable of effectively killing the TMZ-insensitive glioma cells with H2AFJ upregulation provides a feasible strategy for combating the H2AFJ-promoted TMZ resistance in clinical GBM patients.

## Supplementary Materials

The following are available online at https://www.mdpi.com/2072-6694/12/1/98/s1, Figure S1: H2AFJ upregulation is detected in mesenchymal-type GBM tissues, Figure S2: The expression of *H2AFV*, *H2AFX*, *H2AFY* and *H2AFZ* is poorly associated with *IDH1* mutation, *MGMT* promoter methylation and CpG island methylation phenotype (CIMP) in GBM, Figure S3: The expression of *H2AFJ* is associated with age, not gender, of low-grade glioma (LGG)/GBM patients, Figure S4: The pharmaceutical inhibition of HDAC3 and NF-κB re-sensitizes the TMZ-insensitive glioma cells with H2AFJ upregulation to TMZ treatment.
