# Erratum: Lee, H.H., et al. Histone 2A Family Member J Drives Mesenchymal Transition and Temozolomide Resistance in Glioblastoma Multiforme. *Cancers* 2020, *12*, 98

**DOI:** 10.3390/cancers12092649

**Published:** 2020-09-16

**Authors:** Hsun-Hua Lee, Che-Hsuan Lin, Hui-Yu Lin, Chia-Hao Kuei, Jing-Quan Zheng, Yuan-Hung Wang, Long-Sheng Lu, Fei-Peng Lee, Chaur-Jong Hu, Dean Wu, Yuan-Feng Lin

**Affiliations:** 1 Graduate Institute of Clinical Medicine, College of Medicine, Taipei Medical University, Taipei 11031, Taiwan; kaorulei@yahoo.com.tw (H.-H.L.); candycarol0227@gmail.com (H.-Y.L.); pplay1028@gmail.com (C.-H.K.); 16044@s.tmu.edu.tw (J.-Q.Z.); d508091002@tmu.edu.tw (Y.-H.W.); chaurjongh@tmu.edu.tw (C.-J.H.); 2 Department of Neurology, School of Medicine, College of Medicine, Taipei Medical University, Taipei 11031, Taiwan; 3 Dizziness and Balance Disorder Center, Shuang-Ho Hospital, Taipei Medical University, New Taipei City 23561, Taiwan; 4 Department of Neurology, Shuang-Ho Hospital, Taipei Medical University, New Taipei City 23561, Taiwan; 5 Taipei Neuroscience Institute, Taipei Medical University, New Taipei City 23561, Taiwan; 6 Graduate Institute of Medical Sciences, College of Medicine, Taipei Medical University, Taipei 11031, Taiwan; cloudfrank@gmail.com; 7 Department of Otolaryngology, Taipei Medical University Hospital, Taipei Medical University, Taipei 11031, Taiwan; 8 Department of Otolaryngology, School of Medicine, College of Medicine, Taipei Medical University, Taipei 11031, Taiwan; fplee@tmu.edu.tw; 9 Breast Center, Department of General Surgery, Cardinal Tien Hospital, Xindian District, New Taipei City 231, Taiwan; 10 Urology, Division of Surgery, Cardinal Tien Hospital, Xindian District, New Taipei City 231, Taiwan; 11 Department of Critical Care Medicine, Shuang Ho Hospital, Taipei Medical University, New Taipei City 23561, Taiwan; 12 Department of Medical Research, Shuang-Ho Hospital, Taipei Medical University, New Taipei City 23561, Taiwan; 13 Department of Radiation Oncology, Taipei Medical University Hospital, Taipei Medical University, Taipei 11031, Taiwan; lslu@tmu.edu.tw; 14 Graduate Institute of Biomedical Materials and Tissue Engineering, College of Biomedical Engineering, Taipei Medical University, Taipei 11031, Taiwan; 15 Department of Otolaryngology, Shuang-Ho Hospital, Taipei Medical University, New Taipei City 23561, Taiwan; 16 Sleep Center, Shuang-Ho Hospital, Taipei Medical University, New Taipei City 23561, Taiwan; 17 Cell Physiology and Molecular Image Research Center, Wan Fang Hospital, Taipei Medical University, Taipei 116, Taiwan

The authors wish to make the following changes to this paper [1].

The affiliation numbers of the authors Che-Hsuan Lin, Long-Sheng Lu, and Dean Wu are not correct in the original published version. 

The original version is

Hsun-Hua Lee ^1,2,3,4,5^, Che-Hsuan Lin ^6,7^, Hui-Yu Lin ^1,8^, Chia-Hao Kuei ^1,9^, Jing-Quan Zheng ^1,10^, Yuan-Hung Wang ^1,11^, Long-Sheng Lu ^12^, Fei-Peng Lee ^13,14^, Chaur-Jong Hu ^1,2^, Dean Wu ^1,3,4,5,15,^* and Yuan-Feng Lin ^1,16,^*

^1^  Graduate Institute of Clinical Medicine, College of Medicine, Taipei Medical University, Taipei 11031, Taiwan; kaorulei@yahoo.com.tw (H.-H.L.); candycarol0227@gmail.com (H.-Y.L.); pplay1028@gmail.com (C.-H.K.); 16044@s.tmu.edu.tw (J.-Q.Z.); d508091002@tmu.edu.tw (Y.-H.W.); chaurjongh@tmu.edu.tw (C.-J.H.)
^2^  Department of Neurology, School of Medicine, College of Medicine, Taipei Medical University, Taipei 11031, Taiwan
^3^  Dizziness and Balance Disorder Center, Shuang-Ho Hospital, Taipei Medical University, New Taipei City 23561, Taiwan
^4^  Department of Neurology, Shuang-Ho Hospital, Taipei Medical University, New Taipei City 23561, Taiwan
^5^  Taipei Neuroscience Institute, Taipei Medical University, New Taipei City 23561, Taiwan
^6^  Graduate Institute of Medical Sciences, College of Medicine, Taipei Medical University, Taipei 11031, Taiwan; cloudfrank@gmail.com
^7^  Department of Otolaryngology, Taipei Medical University Hospital, Taipei Medical University, Taipei 11031, Taiwan
^8^  Breast Center, Department of General Surgery, Cardinal Tien Hospital, Xindian District, New Taipei City 231, Taiwan
^9^  Urology, Division of Surgery, Cardinal Tien Hospital, Xindian District, New Taipei City 231, Taiwan
^10^  Department of Critical Care Medicine, Shuang Ho Hospital, Taipei Medical University, New Taipei City 23561, Taiwan
^11^  Department of Medical Research, Shuang-Ho Hospital, Taipei Medical University, New Taipei City 23561, Taiwan
^12^  Department of Radiation Oncology, Taipei Medical University Hospital, Taipei Medical University, Taipei 11031, Taiwan; lslu@tmu.edu.tw
^13^  Department of Otolaryngology, Shuang-Ho Hospital, Taipei Medical University, New Taipei City 23561, Taiwan; fplee@tmu.edu.tw
^14^  Department of Otolaryngology, School of Medicine, College of Medicine, Taipei Medical University, Taipei 11031, Taiwan
^15^  Sleep Center, Shuang-Ho Hospital, Taipei Medical University, New Taipei City 23561, Taiwan
^16^  Cell Physiology and Molecular Image Research Center, Wan Fang Hospital, Taipei Medical University, Taipei 116, Taiwan

And should be replaced with

Hsun-Hua Lee ^1,2,3,4,5^, Che-Hsuan Lin ^6,7,8^, Hui-Yu Lin ^1,9^, Chia-Hao Kuei ^1,10^, Jing-Quan Zheng ^1,11^, Yuan-Hung Wang ^1,12^, Long-Sheng Lu ^13,14^, Fei-Peng Lee ^8,15^, Chaur-Jong Hu ^1,2^, Dean Wu ^1,2,4,5,16,^* and Yuan-Feng Lin ^1,17,^*

^1^  Graduate Institute of Clinical Medicine, College of Medicine, Taipei Medical University, Taipei 11031, Taiwan; kaorulei@yahoo.com.tw (H.-H.L.); candycarol0227@gmail.com (H.-Y.L.); pplay1028@gmail.com (C.-H.K.); 16044@s.tmu.edu.tw (J.-Q.Z.); d508091002@tmu.edu.tw (Y.-H.W.); chaurjongh@tmu.edu.tw (C.-J.H.)
^2^  Department of Neurology, School of Medicine, College of Medicine, Taipei Medical University, Taipei 11031, Taiwan
^3^  Dizziness and Balance Disorder Center, Shuang-Ho Hospital, Taipei Medical University, New Taipei City 23561, Taiwan
^4^  Department of Neurology, Shuang-Ho Hospital, Taipei Medical University, New Taipei City 23561, Taiwan
^5^  Taipei Neuroscience Institute, Taipei Medical University, New Taipei City 23561, Taiwan
^6^  Graduate Institute of Medical Sciences, College of Medicine, Taipei Medical University, Taipei 11031, Taiwan; cloudfrank@gmail.com
^7^  Department of Otolaryngology, Taipei Medical University Hospital, Taipei Medical University, Taipei 11031, Taiwan
^8^  Department of Otolaryngology, School of Medicine, College of Medicine, Taipei Medical University, Taipei 11031, Taiwan; fplee@tmu.edu.tw
^9^  Breast Center, Department of General Surgery, Cardinal Tien Hospital, Xindian District, New Taipei City 231, Taiwan
^10^  Urology, Division of Surgery, Cardinal Tien Hospital, Xindian District, New Taipei City 231, Taiwan
^11^  Department of Critical Care Medicine, Shuang Ho Hospital, Taipei Medical University, New Taipei City 23561, Taiwan
^12^  Department of Medical Research, Shuang-Ho Hospital, Taipei Medical University, New Taipei City 23561, Taiwan
^13^  Department of Radiation Oncology, Taipei Medical University Hospital, Taipei Medical University, Taipei 11031, Taiwan; lslu@tmu.edu.tw
^14^  Graduate Institute of Biomedical Materials and Tissue Engineering, College of Biomedical Engineering, Taipei Medical University, Taipei 11031, Taiwan
^15^  Department of Otolaryngology, Shuang-Ho Hospital, Taipei Medical University, New Taipei City 23561, Taiwan
^16^  Sleep Center, Shuang-Ho Hospital, Taipei Medical University, New Taipei City 23561, Taiwan
^17^  Cell Physiology and Molecular Image Research Center, Wan Fang Hospital, Taipei Medical University, Taipei 116, Taiwan

The order of affiliations has been adjusted to make sure they appear in numerical order.

The authors apologize for any inconvenience brought to the readers by these changes. These changes do not affect the scientific results. The manuscript will be updated, and the original will remain online on the article webpage.
