# Supplementary material for: Histone 2A Family Member J Drives Mesenchymal Transition and Temozolomide Resistance in Glioblastoma Multiforme
Source: Cancers (Basel). 2019 Dec 30;12(1):98. doi: 10.3390/cancers12010098 (PMC7016639; doi:10.3390/cancers12010098)
Supplement: Supplementary file 1 [file cancers-12-00098-s001.pdf]

cancers

MDPI

Supplementary Materials

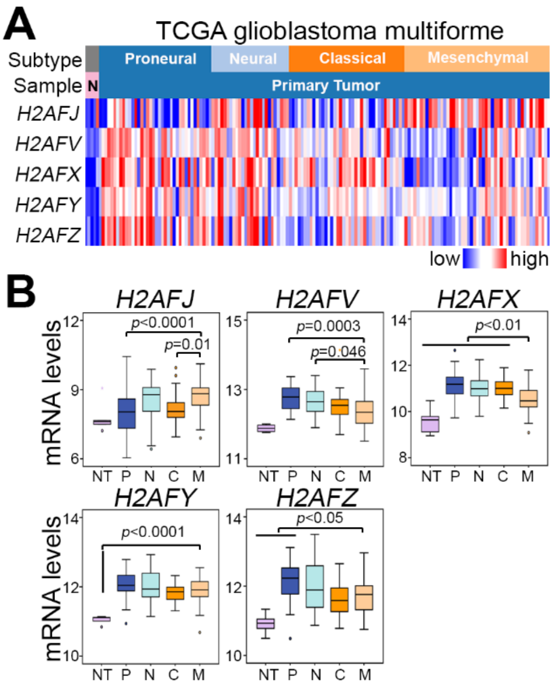

Figure S1. H2AFJ upregulation is detected in mesenchymal-type GBM tissues. (**A** and **B**) Heatmap (**A**) and boxplot (**B**) for the transcriptional profile of the H2A subfamily, which was analyzed by RNA sequencing technique, in normal brain tissues (N for heatmap) and primary tumors derived from patients with different molecular subtypes (proneural, neural, classical and mesenchymal) of GBM using TCGA database. In **B**, statistical significance was estimated by one-way ANOVA and Turkey's post-hoc test.

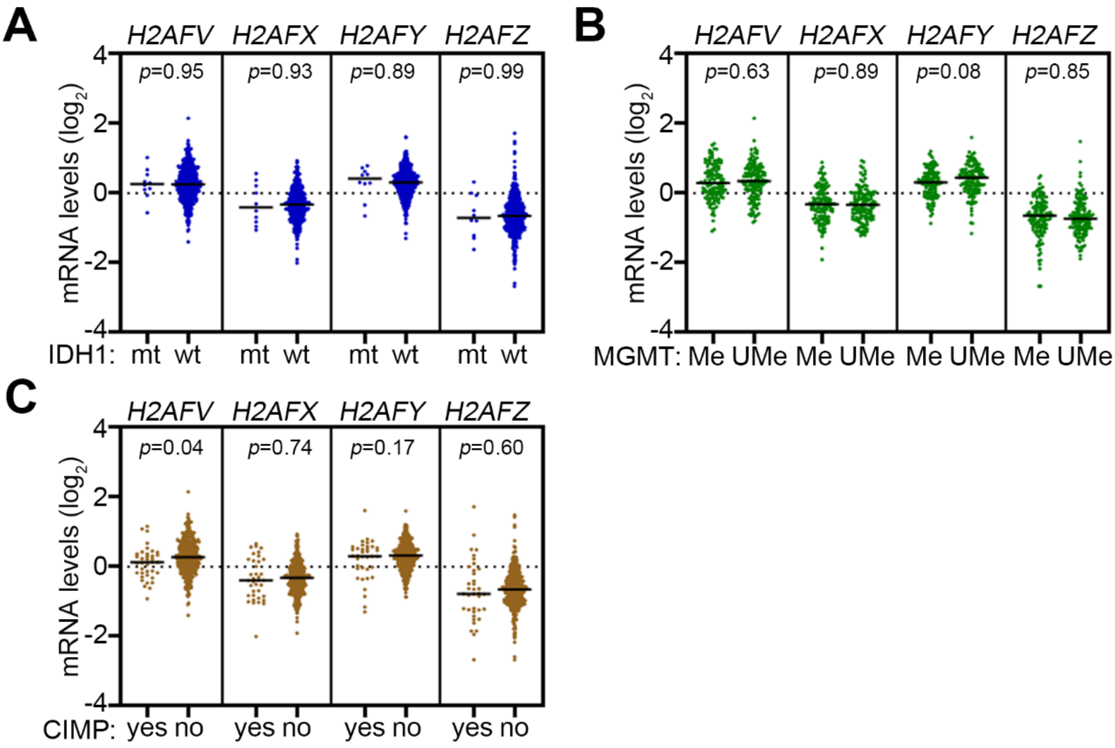

Figure S2. The expression of *H2AFV*, *H2AFX*, *H2AFY* and *H2AFZ* is poorly associated with *IDH1* mutation, *MGMT* promoter methylation and CpG island methylation phenotype (CIMP) in GBM. (**A**–**C**) Dot plots for the transcriptional profiles of *H2AFV*, *H2AFX*, *H2AFY* and *H2AFZ* in *IDH1* mutant and wild-type GBM (**A**), *MGMT* promoter methylated (Me) and unmethylated (Ume) GBM (**B**), or CIMP and non-CIMP-harboring GBM. The statistical significance was determined by Student's t-test.

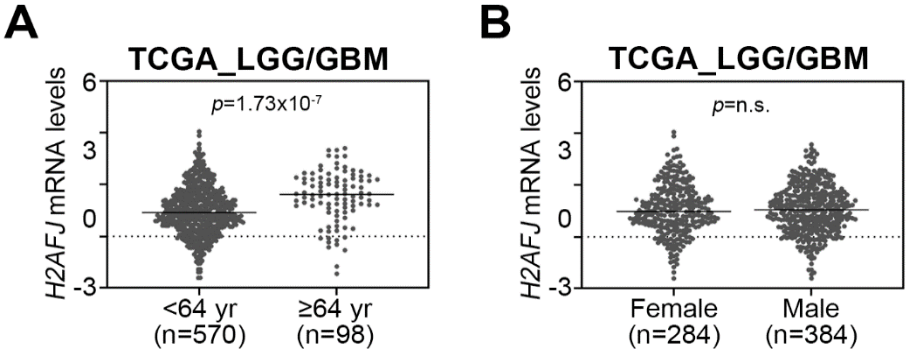

Figure S3. The expression of *H2AFJ* is associated with age, not gender, of low-grade glioma (LGG)/GBM patients. (**A** and **B**) Dot plots for the transcriptional profiles of H2AFJ in primary tumors derived from glioma patients classified by age (**A**) and gender (**B**). The statistical significance was determined by Student's t-test.

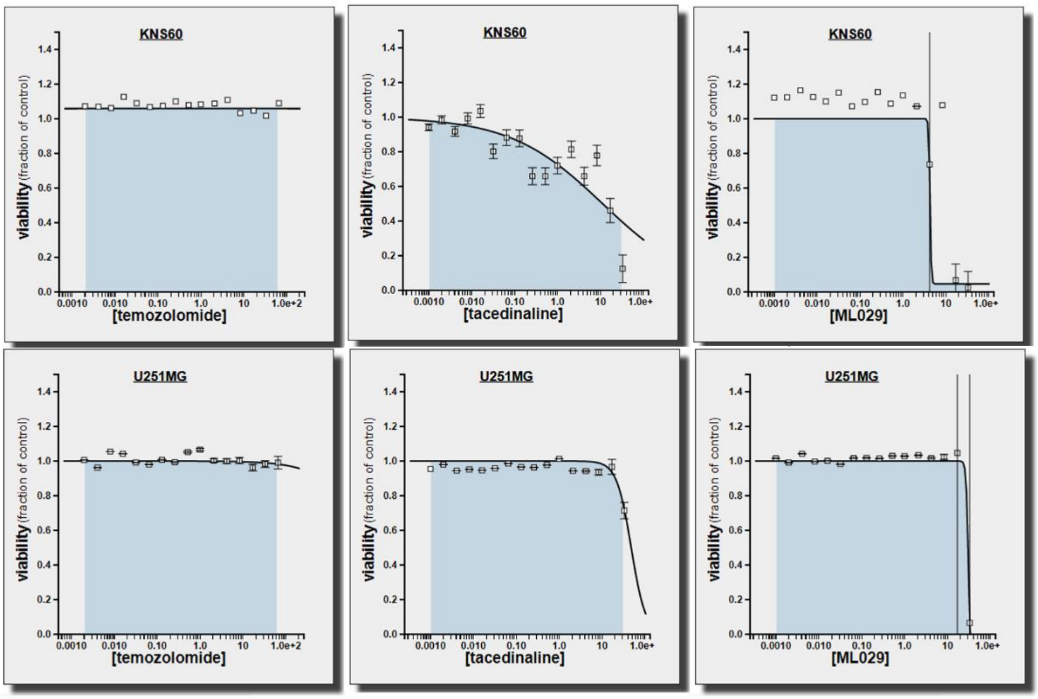

Figure S4. The pharmaceutical inhibition of HDAC3 and NF-κB re-sensitizes the TMZ-insensitive glioma cells with H2AFJ upregulation to TMZ treatment. Scatchard plots for the cell viability of KN560 and U251MG glioma cells in response to the designated concentrations of TMZ, tacedinaline and ML029. Blue area represents the area under the curve (AUC) of receiver operating characteristic curve.
